# The impact of cerebral vasomotor reactivity on cerebrovascular diseases and cognitive impairment

**DOI:** 10.1007/s00702-022-02546-w

**Published:** 2022-10-07

**Authors:** Michela Sforza, Edoardo Bianchini, Diletta Alivernini, Marco Salvetti, Francesco E. Pontieri, Giuliano Sette

**Affiliations:** 1 Dipartimento di Neuroscienze, Salute Mentale e Organi di Senso, Sapienza Università di Roma, Via di Grottarossa, 1035, 00189 Rome, Italy; 2 Azienda Ospedaliero-Universitaria Sant’Andrea, Rome, Italy; 3 Fondazione Santa Lucia, IRCCS, Rome, Italy; 4 INM Neuromed, IRCCS, Pozzilli, IS Italy

**Keywords:** Cerebral vasoreactivity, Cerebrovascular disorders, Cognitive dysfunction, Stroke

## Abstract

The regulation of cerebral blood flow (CBF) is a complex and tightly controlled function ensuring delivery of oxygen and nutrients and removal of metabolic wastes from brain tissue. Cerebral vasoreactivity (CVR) refers to the ability of the nervous system to regulate CBF according to metabolic demands or changes in the microenvironment. This can be assessed through a variety of nuclear medicine and imaging techniques and protocols. Several studies have investigated the association of CVR with physiological and pathological conditions, with particular reference to the relationship with cognitive impairment and cerebrovascular disorders (CVD). A better understanding of the interaction between CVR and cognitive dysfunction in chronic and particularly acute CVD could help improving treatment and rehabilitation strategies in these patients. In this paper, we reviewed current knowledge on CVR alterations in the context of acute and chronic CVD and cognitive dysfunction. Alterations in CVR and hemodynamics have been described in patients with both neurodegenerative and vascular cognitive impairment, and the severity of these alterations seems to correlate with CVR derailment. Furthermore, an increased risk of cognitive impairment progression has been associated with alterations in CVR parameters and hemodynamics. Few studies have investigated these associations in acute cerebrovascular disorders and the results are inconsistent; thus, further research on this topic is encouraged.

## Introduction

Cerebral circulation delivers oxygen and nutrients and removes metabolic products from the brain tissue to ensure neuronal health and brain function. The high metabolic needs of the brain require a large volume of blood flow, comprising almost 20% of the total cardiac output. Therefore, the regulation of cerebral blood flow (CBF) is a complex and tightly controlled function (Aaslid 2006; Wolf 2015; Ashby and Mack 2021). The main mechanisms modulating cerebral hemodynamics, with the aim of adapting CBF to the metabolic demand of the brain, are cerebral autoregulation, vasomotor reactivity and neurovascular coupling (Claassen et al. 2021).

Cerebral autoregulation refers to the intrinsic ability of blood vessels to maintain CBF relatively constant within a wide range of values of systolic blood pressure (50–150 mmHg). Beyond these boundaries, CBF depends passively on perfusion pressure, thus entailing potentially harmful hypo- or hyperperfusion of cerebral tissue (Paulson et al. 1990). Cerebral autoregulation is accomplished through interactions and overlap of three different mechanisms (Wolf 2015): metabolic regulation, mediated by the release of vasoactive substances when oxygen is needed; myogenic regulation, mediated by adaptation of vascular tone to transmural blood pressure; and neurogenic regulation, mediated by sympathetic innervation of the vascular smooth cells. Interactions among these three components have been recently incorporated into the concept of neurovascular unit (NVU), a complex multi-cellular and extra-cellular structure consisting of endothelial cells, neurons, glia, smooth muscle cells, pericytes and extra-cellular matrix. NVU is involved in CBF regulation, blood–brain barrier functioning, immunological surveillance, trophic support and homeostatic cerebral balance. Neurovascular coupling adapts perfusion to increased metabolic demand in response to local changes in neural activity (Kugler et al. 2021) and several studies suggest its key role in signaling, metabolism and brain homeostasis. Impaired autoregulation has been identified in stroke and other neurovascular diseases (Immink et al. 2005), and functional MRI (fMRI) and positron emission tomography (PET) studies contributed at showing the derangement of NVU in patients with hypertension, ischemic stroke and chronic cerebrovascular disease (Wolf 2015).

## Cerebral vasoreactivity

Cerebral vasoreactivity (CVR) refers to the ability of the nervous system to regulate CBF according to the metabolic requirements or chemical variations of the microenvironment (Wolf 2015; Claassen et al. 2021).

The evaluation of CVR is of great interest as an index of "hemodynamic reserve"(HR) in patients with cerebrovascular disease. The HR consists of potential residual vasodilatory capacity plus the possibility of increasing the rate of oxygen extraction from the blood compartment in critical CBF conditions (i.e., cerebral oligemia/ischemia) (Sette et al. 1989). The evaluation of vasoreactivity in a subject is obtained by provoking a transient vasodilatory stimulus and by measuring the variation of CBF. In the presence of a condition of 'maximal vasodilation' (i.e., severe hypoperfusion/oligemia), the flow variation obtained will be minimal or near to zero. Under physiological conditions, the variations in flow obtained are an indication of the entire vasomotor capacity of the subject's hemodynamic reserve.

Arterial pCO_2_ represents a powerful vasomotor stimulus for the resistance arteries of the cerebral circulation. CBF rises in response to increased pCO_2_ and decreased pH, whereas pO_2_ has opposite effects (Smoliński and Członkowska 2016). Small arterioles are extremely sensitive to the vasodilator effect of elevated arterial pCO_2_, with sigmoidal relationship, i.e., linear coupling for pCO_2_ values between 40 and 60 mmHg and non-linear relationship for values outside these limits (Wolf 2015). In particular, the maximal value of vasoreactivity index occurs for pCO_2_ values of 48 mmHg (Claassen 2007) and increases of 1 mmHg of blood pCO_2_ are accompanied by 2.5–5% increased flow rate values in the middle cerebral artery (MCA) compared to basal values (Ide et al. 2003).

Several nuclear medicine and imaging techniques are currently used to measure cerebral hemodynamics and test the response of cerebral vessels to vasoactive stimuli. PET allows direct measuring of CBF and is considered the gold standard to investigate CVR. However, other tools are available, such as near-infrared spectroscopy (NIRS), single photon emission computed tomography (SPECT), fMRI, computerized tomography (CT) with xenon-enhancement, and transcranial doppler sonography (TCD) (Herzig et al. 2008; Rijbroek et al. 2009; Herrera et al. 2016). Despite limited anatomical definition, blood-oxygen-level-dependent (BOLD) fMRI imaging allows rather precise regional specificity, displaying increased signal as the consequence of reduced deoxyhemoglobin concentration (Herrera et al. 2016). TCD is a sufficiently reliable, inexpensive, widely available and non-invasive technique for measuring hemodynamic parameters of main intracranial arteries (Herrera et al. 2016) such as peak systolic velocity (PSV), diastolic velocity (EDV), mean velocity (MV), and pulsatility index (PI) (Bishop et al. 1986; Lindegaard et al. 1987; Valdueza et al. 1999). Despite some intrinsic limitations, TCD with transient vasodilator stimuli has been widely used in clinical practice to assess CVR, reduction of MV indicating decreased global or regional CBF, and high PI pointing on increased microvascular resistance (Chen et al. 2022). Thus, CVR can be estimated by measuring changes of flow velocities in response to vasodilator stimuli in the main cerebral arteries as an indirect indication of changes in CBF (Poulin and Robbins 1996; Bathala et al. 2013). Several studies demonstrated the reliability of TCD in estimating cerebral hemodynamics and CBF variations compared with nuclear and functional imaging (Valdueza et al. 1997; Herrera et al. 2016), but only few reports compared blood flow velocities measured by TCD with CBF measured by PET in patients with symptomatic carotid artery stenosis, with rather variable results (Rijbroek et al. 2009).

In clinical practice, several tests are available to evaluate CVR. The CO_2_ reactivity test measures cerebral vasomotor response after approximately 90-s inhalation of CO_2_ mixtures (3–7%), allowing a simple, non-invasive, reliable and reproducible method (Ringelstein et al. 1992), which requires rather sophisticated technological setting. Acetazolamide test requires intravenous infusion of 500–2000 mg of the carbonic anhydrase inhibitor, acetazolamide, which causes transient, marked, cerebral acidosis and vasodilation (Wolf 2015). This test is widely used for its simplicity and the lack of need of patient’s collaboration; however, it is less accurate and reproducible compared to the former, and it is not devoid of undesirable side-effects such as arterial hypertension, headache, nausea and perioral dysesthesia. Moreover, acetazolamide injection may induce counterproductive hyperventilation, partially neutralizing the vasodilator effect of the drug (Ringelstein et al. 1992; Wolf 2015). Furthermore, standardized examination protocols are lacking for both CO_2_ inhalation and acetazolamide administration. Finally, breath-holding test (BHT) exploits hypercapnia generated by 30-s apnea to calculate a response index defined as breath-holding index (BHI). BHI is a non-invasive, easy to perform, well-tolerated and widely accepted test (Markus and Harrison 1992; Kidwell et al. 2001), despite the limited change of pCO_2_ (approximately 3–4 mmHg), the need for patient collaboration and the reduced reproducibility of this approach.

## Alterations of CVR in aging and neurological disease

Aging is physiologically accompanied by changes of cerebral autoregulation mechanisms with the decrease of CBF and blood volume flow and the reduction of elasticity of small intracranial arteries (Zavoreo et al. 2010). This phenomenon has been associated with vascular changes depending on age-related atherosclerotic processes, which reduce arterial wall flexibility. Moreover, other cardiovascular risk factors such as hypertension, hyperlipemia, diabetes, coronary heart disease and smoking may contribute at reducing capability of cerebral vessels to react at vasodilator stimuli or increased metabolic demand (Gröschel et al. 2007; Staszewski et al. 2021).

Data from the literature show the impairment of CVR in several neurological disorders. Although the role of CVR impairment in the pathogenesis of neurodegenerative disorders has not been defined at present, one can hypothesize that reduced cerebrovascular reserve might further contribute at deteriorating disease progression (Smoliński and Członkowska 2016; Marcic et al. 2021).

In this narrative review, we focused on the impact of CVR on cerebrovascular diseases and cognitive impact. To this end, papers published in English, without publication date limit, were searched in Pubmed and Web of Science databases, using keywords related to: (i) brain circulation (e.g., “cerebral vasoreactivity”, “brain hemodynamics”, “cerebral blood flow”); (ii) cerebrovascular diseases (e.g., “cerebrovascular disease”, “stroke”, “carotid stenosis”, “small vessel disease”, etc.) (iii) cognitive impairement (e.g., “cognitive impairement”, “cognitive decline”, “dementia”, “MCI”, etc.). Search strategy included a combination of keywords, using the Boolean operators “AND” and “OR”. Search fields were restricted to the abstract, title, and keywords. Included papers were original peer-reviewed scientific journal articles, editorials, case studies, letters or reviews. Studies not examining the correlation between CVR, cognitive functions and CVD were excluded. Two independent reviewers [MS and GS] screened the titles and abstracts of all studies to identify potentially relevant articles. Duplicates were manually removed. Full texts of all included studies were then obtained and reviewed. The following 5 parameters were reviewed from the retrieved articles by two independent reviewers: (1) study characteristics; (2) participant characteristics; (3) tools for measuring CVR; (4) measure of cognitive function; and (5) main findings. Quantitative analysis and assessment of the risk of bias were not performed due to the narrative design of the study. The quality assessment was appraised by two independent reviewers (MS, EB); disagreements between evaluators were resolved through discussion.

## CVR and cognitive impairment

Several studies demonstrated the correlation between alteration of brain vessel reactivity and impaired cognitive functions in patients with cognitive derangement. Arteriolosclerosis, amyloid angiopathy, atherosclerosis and lipohyalinosis have been associated with impaired cerebral hemodynamic and cognitive impairment (Chen et al. 2022). Alterations of cerebral vessel resistance using TCD, in particular the PI, and evaluation of CVR to hypercapnia using BHI have been consistently associated with cognitive decline and are indicative of cognitive impairment in Alzheimer’s disease (AD) and vascular dementia (VaD) (Keage et al. 2012). Several authors investigated the possible correlation between altered cerebral hemodynamic and cognitive functions in mild cognitive impairment (MCI) or AD. In general, low intracranial arterial MVF, increased vascular resistance (PI) and reduced vasoreactivity have been identified in these pathological conditions with respect to healthy subjects (Silvestrini et al. 2006; Lim et al. 2018; Cipollini et al. 2019; Chen et al. 2022). Moreover, it has been suggested that TCD may predict clinical progression of cognitive decline, higher PI and lower BHI in the middle cerebral artery being associated with stronger risk of conversion to dementia in subjects with MCI, with significant correlation between decreased BHI and reduced MoCA score (Silvestrini et al. 2006; Lim et al. 2018). Eventually, TCD may help differentiating individuals with MCI from healthy subjects (Zavoreo et al. 2010; Chung et al. 2017). Taken together, these data suggest that early cerebral microvascular abnormalities in the brain may anticipate the occurrence of significant cognitive impairment, and possibly precede the development of structural brain lesions identifiable on conventional imaging (Chen et al. 2022).

Several other factors have been shown to play a role along with CVR impairment in cognitive decline, including aging, altered brain energy demand, and hypoperfusion. Detailed analysis of these factors is beyond the scope of this work, exhaustive reviews on the relationships between hypoperfusion, brain energy demand and cognitive decline being available in the recent literature (Popa-Wagner et al. 2014, 2015; Ciacciarelli et al. 2020).

## CVR in cerebrovascular diseases

Most studies investigating the relationships between impaired cerebral hemodynamic, CVR and cognitive impairment were carried out on subjects affected by cerebrovascular disorders. Research focused on presymptomatic subjects (carotid stenosis) (Silvestrini et al. 1996; Silvestrini et al. 2000; Markus and Cullinane 2001; Cheng et al. 2012; Zavoreo et al. 2013; Viticchi et al. 2021), acute ischemic stroke (Alvarez et al. 2004; Uzuner et al. 2013; Salinet et al. 2015, 2019; Altmann et al. 2016; Chi et al. 2020) and chronic cerebrovascular disorders (Provinciali et al. 1990; Matteis et al. 1998; Kidwell et al. 2001; Shiogai et al. 2002; Sabayan et al. 2012; Turk et al. 2016; Kisler et al. 2017; Bian et al. 2019; Staszewski et al. 2021).

### Carotid stenosis

Cerebral perfusion is rather variable in subjects affected by carotid occlusive disease, and appears related to the degree of collateral blood supply rather than severity of stenosis (Silvestrini et al. 2000). Many subjects with steno-occlusive carotid disease develop compensatory vasodilation of ipsilateral arteries together with collateral circulation, and patients with high grade carotid stenosis display reduced CBF in the ipsilateral brain hemisphere, particularly in MCA borderline regions, that may revert towards normal values after revascularization therapy (Schroder et al. 2019). In these subjects, further vasodilator stimulus by hypercapnia will produce absent or markedly reduced vasodilator response (Silvestrini et al. 1996). The reduction of CVR is associated with increased risk of ipsilateral stroke or TIA in patients with carotid occlusion and, to a lesser extent, asymptomatic carotid stenosis (Viticchi et al. 2021). This suggests that assessment of CVR may help identifying high-risk patients who may benefit from revascularization.

There is also clear evidence for the association between carotid stenosis and the onset and progression of cognitive impairment, even in subjects with severe asymptomatic carotid stenosis (Cheng et al. 2012; Zavoreo et al. 2013; Viticchi et al. 2021), as if carotid stenosis might facilitate cognitive dysfunction through the combination of increased incidence of acute cerebrovascular lesions, microembolization, chronic hypoperfusion, and impairment of CVR (Viticchi et al. 2021). Indeed, impaired cerebral hemodynamics may lead to altered regional functional connectivity, particularly in the fronto-parietal network, in turn inducing cognitive dysfunction (Cheng et al. 2012).

Consequently, revascularization procedures have been hypothesized recently as a way to restore hemodynamically induced cognitive impairment. Further randomized clinical trials on large cohorts are required, however, to define the effectiveness and timing of medical and surgical/endovascular approaches to this issue (Viticchi et al. 2021).

### Acute ischemic stroke

Cerebral hemodynamic impairment plays a significant pathophysiological role in the acute phase of cerebral ischemia, and somehow predicts stroke severity, progression and long-term outcome. The correlation between impairment of CVR and the occurrence of acute ischemic stroke in patients with severe internal carotid artery stenosis has been convincingly confirmed (Gur et al. 1996; Silvestrini et al. 1996, 2000; Markus and Cullinane 2001; Cheng et al. 2012; Viticchi et al. 2021). However, the role of altered CVR in patients with acute ischemic stroke without significant carotid stenosis has not been clarified at present.

Hemodynamic factors and cerebral hemodynamic reserve have been related to the final infarct volume, unfavorable long-term outcome and most neurologic complications after acute stroke (Alvarez et al. 2004). Thus, cerebral hemodynamic parameters are progressively compromised according to stroke severity: moderate and severe stroke are accompanied by greater CBF asymmetries between the affected and unaffected hemisphere, derangement of autoregulation mechanisms in the affected hemisphere, and bilateral NVU impairment (Salinet et al. 2019). Furthermore, studies demonstrated depressed CBF response to neural activation and CO_2_ (Salinet et al. 2015), and impaired vasoreactivity appears coupled with poorer functional outcome (Salinet et al. 2019). Other reports showed the association between cerebral hemodynamic alterations and cognitive performances in patients with acute cerebral ischemia. In the case of lacunar infarcts, the increase in PI has been correlated to impairment of executive functions (Sivakumar et al. 2017). The increase of PI measured proximally to the blood vessels reflects the increased distal vascular resistance and consequent reduction of diastolic flow that may depend on alterations of microcirculation secondary to subcortical ischemic events (Uzuner et al. 2013). With this respect, studies on animal models showed the dysfunction of capillary pericytes as the consequence of oxygen radical production in ischemia (Yemisci et al. 2009). This may lead to vascular contraction and hypoperfusion, in turn causing functional alterations of connectivity within associative networks (Chi et al. 2020). A recent pilot study, however, did not support such hypothesis, and suggested that hemodynamic alterations may contribute at worsening cognitive performances transiently, during the first 3–6 months following acute subcortical ischemia (Suministrado et al. 2017).

Eventually, animal studies and neuropathological findings in humans confirm that relevant angiogenesis occurs in post-stroke brain tissue (Buga et al. 2014). The impact of post-stroke angiogenesis on CVR has not been defined completely at present, despite the theoretical observation of partial increase of the cerebrovascular bed. Future studies are advised to identify whether these newly generated vessels develop normal response to stimuli modulating CVR.

### Chronic cerebrovascular disorders

A conspicuous body of research focused on the association between impaired CVR and cerebral small vessel disease (SVD). Microangiopathy represents a prominent cause of lacunar stroke and vascular dementia (VaD) (Staszewski et al. 2021). SVD is a dynamic and progressive pathology involving several components of the NVU, generating dysfunction of signaling pathways that may be responsible for CBF deregulation in VaD (Kisler et al. 2017). TCD with BHI showed the reduction of CVR in patients with SVD with respect to control subjects (Staszewski et al. 2021). Moreover, the results of meta-analyses demonstrated that alteration of cerebral hemodynamic in VD patients is more pronounced that AD (Sabayan et al. 2012). Thus, patients with SVD and chronic cerebrovascular pathology display increased rate of blood flow and higher PI in the larger intracranial arteries, which correlate with the severity of cognitive impairment, the vascular Hachinski scale score, and the degree of leukoaraiosis at MRI (Kidwell et al. 2001; Turk et al. 2016). Kindwell and collaborators (2001) also showed that the PI is an independent predictor of SVD, with specificity and sensitivity values of 89% and 86% for periventricular hyperintensities and 70% and 73% for deep matter hyperintensities. In addition, other studies showed the association between multi-infarct leukoencephalopathy and CVR alteration as measured by acetazolamide test (Shiogai et al. 2002) or in response to apnea (Provinciali et al. 1990; Matteis et al. 1998). In particular, reduction of CVR as measured by BHI seems directly associated with the severity of leukoaraiosis, and patients with lower BHI and moderate-to-severe SVD (grade 2–3 at the Fazekas scale) display lower performances at the MoCA as well as other tests measuring executive functions (Turk et al. 2016; Bian et al. 2019). BHI might, therefore, be helpful for evaluating the alterations of CVR in subjects suffering from leukoaraiosis, and represent an indicator of cognitive dysfunction in these patients.

Obstructive sleep apnea syndrome (OSAS) is a frequent disease in aging and a well-described condition associated with CVR impairment. OSAS is a disorder characterized by recurring episodes of obstruction and partial or complete collapse of the rhino‐oropharynx during sleep. These are caused by anatomical upper airway alteration combined with impaired ventilatory control or alteration of neurofunctional control of rhino- and oropharyngeal muscles. The episodes lead to intermittent oxygen desaturation and are associated with sleep fragmentation, cerebrovascular and cardiovascular disease, excessive daytime sleepiness and cognitive dysfunction. A comprehensive review by Beaudin et al. (2017) addressed the impact of OSA on cardiovascular and cerebrovascular regulation. A more recent comparative study carried out by our group (Piraino et al. 2019) on 40 patients suffering from moderate-to-severe OSAS (AHI ≥ 15) showed the tendency to increased BHI and the significant reduction of IMT after continuous positive airway pressure (CPAP) treatment, therefore suggesting that CPAP treatment may improve CVR and reduce endothelial inflammation.

## Association between CVR and brain regions and functions

In recent years, evidence has accumulated on the association between impaired cerebral hemodynamics and cognitive functions in patients suffering from neurological disorders, in particular cerebrovascular diseases, as if reduction of cerebral blood flow and brain perfusion may contribute to cognitive derangement (Mori et al. 1994; Firbank et al. 2011). Dementia associated with CVD is rather common, affecting 25–30% of elderly stroke survivors as post-stroke dementia (PSD), which frequently meets criteria for VaD (Allan et al. 2011). Cognitive impairment in VaD is frequently sustained by damage of the frontal–subcortical circuits (Kalaria and Ihara 2013). The frontal lobe is particularly vulnerable to vascular-based pathology (Jobson et al. 2021), and previous studies reported that 50% of stroke survivors display deficits of executive functions, regardless the severity or subtype of cerebrovascular event. A further potential link for developing PSD is the preferential location in the frontal lobe of white matter vascular pathology supported by astrogliosis or clasmatodendritic changes in microvessel with irreversible astrocyte injury, disruption of gliovascular interactions and blood–brain barrier (Chen et al. 2016). Previous studies showed that medullary arteries and the telencephalic white matter of the frontal lobe are particularly susceptible to cerebral hemodynamic disturbance (Ihara et al. 2010), and that a correlation exists between reduced cerebral blood flow and these pathological changes, as if cerebral hypoperfusion might represent a direct cause of vascular pathology (Qin et al. 2010) in particular in elderly subjects. Indeed, there is evidence that clasmatodendrosis occurs acutely after induction of cerebral hypoperfusion in non-human primates (Chen et al. 2016), and that impairment of cognitive performances within the first three months post-stroke might predict future recovery (Park et al. 2015).

Executive functions are essential for regulating goal-oriented behaviors and responding to new and novel settings, by combining working memory, planning, orientation problem-solving, self-monitoring and error correcting. Recent studies suggest that executive dysfunction in stroke patients produces reduced performances in both basic and complex activities of daily living and compromises rehabilitation outcomes (Zinn et al. 2007; Chung et al. 2013). Therefore, one can speculate that stroke outcome and post-rehabilitation improvement might benefit from precocious identification and management of executive dysfunction as well as by rapid therapeutic management by compensatory and stimulating approaches (Zinn et al. 2007).

## Conclusions and perspectives

Table 1 summarizes the main characteristics of cerebral vasomotor reactivity in aging, cerebrovascular disease and cognitive impairment. In conclusion, the relationship between impaired cerebral hemodynamics, in particular CVR, and executive functions has been clearly defined in chronic cerebrovascular disease subjects but is rather limited in acute stroke patients. Future research in this field should be focused on defining the interactions between impaired cerebral hemodynamic parameters and executive functions in the acute phase of cerebral ischemia, and the potential predictive role of these changes on long-term functional outcomes after acute ischemic stroke. Thus, cerebral hemodynamic should be monitored over time following acute ischemic stroke to investigate its possible predictive role on response to neurorehabilitation and long-term functional outcome. Eventually, differences in cerebral hemodynamic changes should be investigated with respect to location and severity of acute stroke, to plan personalized therapeutic strategies, and the relationships between impaired hemodynamic parameters and other cardiovascular risk factors on cognitive outcome should be defined. This would allow the results of experimental and human studies to be translated into clinical practice and help select the best parameters to identify high-risk patients, predict functional outcome, and monitor patients over time.

**Table 1:** Summary of main characteristics of cerebral vasomotor reactivity in aging, cerebrovascular disease and cognitive impairment

Neurological condition	Alteration of hemodynamic parameters	Conclusions
Aging	Decreasing trend in CBF due to aging and good correlation between the decreasing trend in BHI values and MoCA score in older patients (Zavoreo et al. 2010) CBFV and CVR changes were significantly lower in the group of old subjects with vascular risk factors compared with the healthy young and old subjects (Gröschel et al. 2007)	Age-related atherosclerotic processes and other cardiovascular risk factors is accompanied by changes of cerebral autoregulation mechanisms with the decrease of CBF and the reduction of elasticity of small intracranial arteries with reduction of capability of cerebral vessels to react at vasodilatory stimuli
MCI and Alzheimer disease	Alterations of PI and BHI have been consistently associated with cognitive impairment in AD (Keage et al. 2012) Low intracranial arterial MVF, increased PI and reduced CVR have been identified in MCI and AD with respect to healthy subjects (Silvestrini et al. 2006; Lim et al. 2018; Cipollini et al. 2019; Chen et al. 2022); Higher PI and lower BHI in the MCA being associated with stronger risk of conversion to dementia in subjects with MCI (Silvestrini et al. 2006; Lim et al. 2018)	TCD with evaluation of CBF and CVR may predict clinical progression of cognitive decline and may help differentiating individuals with MCI from healthy subjects
Vascular dementia and small vessel disease	Patients with SVD and chronic cerebrovascular pathology display increased CBF, higher PI and reduction of BHI in the larger intracranial arteries, which correlate with the severity of cognitive impairment, vascular Hachinski scale score, degree of leukoaraiosis at MRI and low performance at cognitive tasks (Turk et al. 2016; Bian et al. 2019; Kidwell et al. 2001; Staszewski et al. 2021; Shiogai et al. 2002; Provinciali et al. 1990; Matteis et al. 1998)	More studies showed the association between multi-infarct leukoencephalopathy and CVR alteration BHI might be helpful for evaluating the alterations of CVR in subjects suffering from leukoaraiosis, and represent an indicator of cognitive dysfunction in these patients
Asymptomatic carotid stenosis	In subjects suffering from severe asymptomatic carotid stenosis, altered CVR has negative prognostic value, and the reduction of BHI ipsilateral to stenosis strongly increases the risk of cerebrovascular ischemic events (Silvestrini et al. 2000; Viticchi et al. 2021) Patients with high grade carotid stenosis present alterations of brain perfusion in the hemisphere of the stenosis. This changes are reversible after revascularization therapy (Schroder et al. 2019) Impaired cerebral hemodynamics may lead to altered regional functional connectivity, particularly in the fronto-parietal network, in turn inducing cognitive dysfunction (Cheng et al. 2012)	Carotid atherosclerosis has a relevant impact on cerebral blood flow regulation. In the last years an increasing number of findings showed that carotid stenosis did contribute to cognitive impairment not only in relation to the occurrence of cerebral ischemic lesions, but also as an independent risk factor. The principal mechanisms involved are chronic hypoperfusion, microembolization and cerebrovascular reactivity impairment. Therefore, assessment of CVR may help identifying high-risk patients who may benefit from revascularization (Shroder et al. 2019)
Acute ischemic stroke	The correlation between impairment of CVR and the occurrence of acute ischemic stroke in patients with severe internal carotid artery stenosis has been convincingly confirmed (Gur et al. 1996; Silvestrini et al. 1996; Silvestrini et al. 2000; Markus and Cullinane 2001; Cheng et al. 2012; Viticchi et al. 2021) Alterations of hemodynamic factors and CVR have been related to the final infarct volume, unfavorable long-term outcome and most neurologic complications after acute stroke (Alvarez et al. 2004) Cerebral hemodynamic parameters are progressively compromised according to stroke severity (Salinet et al. 2019) In lacunar infarcts, the increase in PI has been correlated with impairment of executive functions (Sivakumar et al. 2017) A recent pilot study and suggested that hemodynamic alterations may contribute at worsening cognitive performances transiently, during the first 3–6 months following acute subcortical ischemia (Suministrado et al. 2017)	Cerebral hemodynamic impairment plays a significant pathophysiological role in the acute phase of cerebral ischemia, and somehow predicts stroke severity, progression and long-term outcome

## Abbreviations

AD: Alzheimer’s disease
BHI: Breath-holding index
BHT: Breath-holding test
CBF: Cerebral blood flow
CT: Computerized tomography
CVD: Cerebrovascular disorders
CVR: Cerebral vasoreactivity
EDV: End diastolic velocity
fMRI: Functional MRI
MCA: Middle cerebral artery
MCI: Mild cognitive impairment
MVF: Mean velocity flow
NIRS: Near-infrared spectroscopy
NVU: Neurovascular unit
OSAS: Obstructive sleep apnea syndrome
PET: Positron emission tomography
PI: Pulsatility index
PSD: Post-stroke dementia
PSV: Peak systolic velocity
PECT: Photon emission computed tomography
SVD: Small vessel disease
TCD: Transcranial Doppler
VD: Vascular dementia
